# Current evidence and future directions for social and societal resilience factors in response to societal challenges and crises: an overview of systematic reviews and expert rating

**DOI:** 10.1186/s12889-025-25285-5

**Published:** 2025-11-06

**Authors:** Max Supke, Lea M. Schaubruch, Caroline Cohrdes, Corinna Kausmann, Sarah K. Schäfer, Klaus Lieb

**Affiliations:** 1 Leibniz Institute for Resilience Research, Wallstraße 7, Mainz, 55122 Germany; 2 Department of Clinical Psychology, Psychotherapy and Psychodiagnostics, Technische Universität Braunschweig, Braunschweig, Germany; 3 Department of Epidemiology and Health Monitoring, Robert Koch Institute, Berlin, Germany; 4 Department of Psychiatry and Psychotherapy, University Medical Center of Johannes Gutenberg University, Mainz, Germany

**Keywords:** Social and societal resilience factors, Overview of systematic reviews, Adults, Societal crises, Mental health, Expert survey

## Abstract

Resilient outcomes are the most common response to societal crises. Resilience factors represent multilevel psychosocial resources that increase the likelihood of resilient responses. While resilience research has predominantly focused on individual factors, social and societal factors have received less attention. This overview of systematic reviews aimed to identify and summarize evidence on social and societal resilience factors linked to mental health responses during societal challenges in high-income countries. Systematic reviews exploring these associations were identified through searches in three databases up to June 12, 2023. Resilience factors were classified as having favorable, unfavorable, mixed, or non-significant associations with mental health outcomes. Quality appraisal used an adapted AMSTAR-2-tool. Twenty reviews (samples: 2,402–489,419) reported on stressors like pandemics, minority stress, and migration. At the social level, stronger evidence supports the favorable effects of social support, relationship quality, and family support. Societal factors like adequate housing, access to mental health services, and supportive policies were linked to resilience outcomes, though evidence remains sparse or heterogenous for many factors. An expert survey rated social factors as slightly more important than societal ones. Expanding resilience research to include families, workplaces, and societal factors in longitudinal studies will enhance the understanding of resilience from a systemic perspective.

**Supplementary Information:**

The online version contains supplementary material available at 10.1186/s12889-025-25285-5.

## Introduction

Resilience, when defined from an outcome-based perspective, refers to the maintenance or quickly recovery of mental health during or after exposure to stressors. A resilient *trajectory* is characterized by an at most brief period of psychological disequilibrium following adverse life events or challenging circumstances, with mental health otherwise remaining stable [1–3].

This outcome-based approach to resilience research focuses on the trajectories of mental distress and positive mental health in response to stressors [4–7]. Despite the psychological burden typically associated with potentially traumatic events, systematic reviews and meta-analyses conclude that approximately 66% of affected individuals demonstrate *resilient* responses. These responses are characterized by consistently low levels of mental distress or the maintenance of good mental health despite exposure to stressors. In contrast, approximately 11% of individuals show *chronic* symptoms, exhibiting persistently high levels of mental distress. Additionally, 13–21% of the affected people follow a *recovery* trajectory, characterized by an initial increase in distress followed by a gradual decrease over time. A smaller subset of all the affected individuals (9–12%) display a *delayed* response, initially experiencing low levels of distress, followed by a subsequent increase. Importantly, the severity of the potentially traumatic event does not seem to significantly influence the distribution of distinct trajectories, as even more severe events (e.g., displacement after natural disaster or police officers exposed to life threat) are associated with high rates of resilient trajectories. This could suggest that psychological, social, and biological factors may play a key role in helping people maintain or regain their mental health even in the face of more severe stressors [7, 8].

The concept of resilience has gained increasing attention as societal challenges, crises, and stressors become more frequent. Many nations are currently confronted with a multitude of societal crises like wars and armed conflicts, the climate crisis, the COVID-19 pandemic, economic crises, and various natural disasters [9, 10]. These crises are often accompanied by displacement or significant disruptions of living conditions for large populations, which in turn elevate stress levels and increase the risk of mental health disorders [11–13]. Against this background, the question of how individuals manage to maintain or quickly restore their mental health in the face of such challenges has gained importance [14].

This ties in with the societal challenge of how individuals can be supported in responding resiliently to stressor exposure. Ungar and Theron [15] propose that resilience is shaped by the dynamic interplay of biological, psychological, social, and ecological systems throughout the life course of an individual. Within this context, so-called *resilience factors* become crucial. Adopting a multisystemic approach based on Ungar and Theron [15], resilience factors can be categorized into individual, social, and societal domains. Resilience factors are multilevel resources that have the potential to protect individuals from harmful effects of stressors and thereby increase the likelihood of resilient responses [16, 17]. While resilience research often prioritizes internal, individual factors – such as optimism, sense of coherence, and beliefs about perceived control – less attention is given to the social, cultural, and societal systems that shape individuals’ environments [15, 18].

The predictive power of individual resilience factors remains inconclusive [19–21], as some may not affect stress responses at all, and others may even correlate positively with mental distress. Moreover, individual factors often diminish in significance when multiple factors are considered in the same statistical model for the prediction of resilient outcomes. However, neither individual nor combined factors serve as reliable predictors of resilient responses. Another challenge is the lack of conceptual clarity, as there is significant empirical and conceptual overlap among different individual resilience factors (e.g., self-efficacy and locus of control; meaning in life and spirituality; [8, 22]).

Human development is shaped by the complex interplay between individuals and their surrounding environments. Ecological theories differentiate between four distinct layers, which differ in terms of how close they are to the individual [23, 24]. The closest layer, the *microsystem*, includes factors such as personal characteristics and emotions (examples for resilience factors in this layer are optimism [25] or self-efficacy beliefs [22, 26]). The *mesosystem* encompasses elements like intimate partner relationships (e.g., relationship quality, being married). The *exosystem* extends further and includes factors such as family relationships (e.g., family support) that are outside the immediate sphere of the individual. Finally, the *macrosystem*, the most distant level, encompasses systemic and societal factors (e.g., trust in institutions; [23, 24]). Many studies analyzing societal challenges and crises primarily include factors at the microsystem level into their models, while factors at the macrosystem level (such as those related to broader societal or cultural systems and environments) are rarely evaluated as potential resilience factors. Considering social and societal factors and incorporating them into statistical models may improve the accuracy of resilience predictions. For example, Hobfoll et al. [17] highlight that resilience is strongly influenced by a supportive environment and the presence of secure, loving relationships, suggesting that these factors play a critical role.

In this present overview of systematic reviews, we define *social resilience factors* (e.g. factors from the meso- and exosystem) as perceived and available resources within one’s immediate social environment, including elements such as social support, family cohesion, having a partner, and one’s social network size (i.e., structural social support). At the social level, psychological and public health research frequently examined received and perceived social support, which is generally associated with better mental health and more resilient outcomes [27–29]. While there is considerable research on the role of social support, and at times an overemphasis on it, other potential social resilience factors are often overlooked.

*Societal resilience factors* (e.g., factors from the macrosystem) refer to resources that are perceived or available at a broader societal level, such as access to green spaces (e.g., parks), blue environments (e.g., lakes), community cohesion, job security, and trust in institutions. These factors have been less frequently studied, and the current state of research on them remains very sparse [30–32].

Arcaya, Raker, and Waters [12] stress the importance of social and societal factors in supporting recovery after disasters. They note that community cohesion, social capital, and economic investment play key roles in either helping areas recover or leading them toward decline. Communities recover from crises to varying degrees and with varying durations, influenced by factors such as socioeconomic status, support from external sources, and prior experiences with disasters [30, 33, 34]. Social cohesion, which includes connectedness and solidarity among different community groups, also plays a critical role in recovery from crises, such as the COVID-19 pandemic [31].

The current project was thematically inspired by a recent systematic review of our research team [27] that summarized the evidence on the predictive value of individual, social, and societal resilience factors in the context of societal challenges and crises (e.g., pandemics, economic crises, natural disasters). This systematic review synthesized findings from 50 primary studies using growth mixture modeling to study post-stressor mental health, revealing that higher income, higher socioeconomic status, greater perceived social support, better emotion regulation, and higher psychological flexibility were associated with more resilient responses following such crises. Yet, most effect estimates were small, and results were mixed for many resilience factors. Notably, the majority of studies focused primarily on individual and social resilience factors (i.e., perceived social support), with fewer studies incorporating social factors other than social support and societal resilience factors into their models. For the described review, we focused on primary studies examining resilient responses in face of stressor exposure by means of growth mixture modeling [5, 6], a specific methodological approach to study resilience as an outcome. However, it remained unclear what is known of social and societal resilience factors based on a larger body of evidence using more heterogeneous methodological approaches.

### Objectives

For this purpose, the current overview of systematic reviews aimed at summarizing evidence on social and societal resilience factors at the level of systematic reviews. The inclusion of a broader range of social and societal factors could enhance the understanding of mental health trajectories following societal crises and challenges. This overview of systematic reviews tried to address this gap and tried to contribute to a more comprehensive understanding of how various social and societal resilience factors impact mental health outcomes in the face of societal disruptions. We focused on societal crises and challenges in OECD member states which are predominantly high-income countries and characterize themselves as democracies [35]. These commonalities provided a baseline level of comparability, enabling more consistent and meaningful analyses.

This overview of systematic reviews aimed to address the following research questions: (1) Which societal challenges and crises have been examined in the included systematic reviews? (2) What types of mental health outcomes have been studied in relation to these challenges? (3) Which social and societal resilience factors have been examined as correlates of these mental health outcomes? (4) What associations (favorable, unfavorable, non-significant, or mixed) between resilience factors and mental health outcomes have been observed? (5) What social and societal factors do experts in the domains of resilience research, public health, and family studies consider to be important? Based on these findings, we derive potential future directions and recommendations for resilience research.

## Methods

### Protocol and Pre-Registration

This systematic overview of systematic reviews followed the checklist for overviews of systematic reviews developed by Onishi and Furukawa ([36]; see Supplementary Material 1). It is part of a larger project titled “A Systematic Review of Individual, Social, and Societal Resilience Factors in Response to Societal Challenges and Crises” which summarizes evidence on the predictive value of individual, social, and societal resilience factors on mental health outcomes during societal crises [27]. During the data extraction process for the systematic review in the main project, it became apparent that there is a lack of studies systematically examining a broad range of social and societal resilience factors. This research gap was the starting point of this subproject. The overall project was prospectively preregistered (https://osf.io/gwjva), and the study protocol for the main project is available on the Open Science Framework (https://osf.io/9xwyu/). The data for this overview of systematic reviews is available at https://osf.io/f4zty/.

### Search strategy

Three databases were searched from 2013 to present (last update: June 12, 2023), including *Embase* (incl. PubMed and EmbaseCore), *Scopus*, and *Web of Science Core Collection*. The primary search contained two clusters with search terms related to (1) the social and societal level (e.g., social, societal, community, family), and (2) resilience factors and psychosocial resources (e.g., resilience factor, protective factor, health-promoting factor). Terms within one cluster were linked using the Boolean operator *OR* and clusters were combined using the operator *AND* (see Supplementary Material 2 for the full search strategies). Furthermore, scientific discussions and interviews were conducted with experts in the fields of public health and family psychology to identify key systematic reviews and relevant resilience factors. Additionally, reference lists of the included systematic reviews were checked for eligible studies.

### Inclusion criteria

Eligible studies for this overview of systematic reviews were systematic reviews (which could include meta-analyses) that met the following criteria: (a) The systematic review focused primarily on adult individuals (≥ 18 years) from the (b) general civilian population, not recruited from military or clinical contexts, (c) who were exposed to various societal challenges and crises, (d) in OECD member countries (at least 66% of the included primary studies in the systematic reviews needed to be conducted in OECD countries). Such stressors included pandemics, wars, armed conflicts, the climate crisis, migration, experienced minority stress, and natural disasters as well as man-made disasters. (e) The reviews had to report on social and/or societal resilience factors (i.e., any factors discussed as potentially promoting health or resilience; [2, 22, 37, 38]), with no predefined list of resilience factors being applied for this review. (f) Additionally, associations between resilience factors and mental health outcomes, whether related to mental distress (e.g., depression, anxiety, substance use disorder) or positive mental health (e.g., quality of life, well-being), needed to be reported.

### Study selection

After de-duplication with Zotero [39], two reviewers (FS and IW) independently screened titles, abstracts, and full texts using Rayyan [40]. Interrater reliability was substantial, with kappa values of 0.66 for the title/abstract screening and 0.86 for the full-text review. Discrepancies at both stages were resolved through discussion or, when necessary, by consulting a senior team member (MS/SKS).

### Data extraction

A customized data extraction sheet was developed for this overview of systematic reviews and is available via the OSF project associated with this review (https://osf.io/f4zty/). Data from all eligible primary studies were extracted by one reviewer and checked by a second reviewer (FS/IW). Any discrepancies were resolved through discussion or by consulting senior team members (MS/SKS). Data extraction focused on review characteristics, sample demographics, types of societal challenges, resilience factors, mental health outcomes, and the evidence rating for each resilience factor. Additionally, information required for subsequent quality appraisal was extracted.

The main objective of this overview of systematic reviews was to compile a comprehensive collection of social and societal resilience factors in the context of societal crises and challenges, as identified from systematic reviews. Resilience factors were classified as either social or societal resources by one reviewer (MS), and this classification was checked by a second reviewer (SKS). Any disagreements were discussed and resolved within the review team. Social factors were defined as resources perceived or available in one’s proximal social environment (e.g., family, friends, work colleagues), whereas societal factors referred to resources in the broader societal context (e.g., policies, culture, living environment). The associations between these resilience factors and mental health outcomes were extracted. Data were collected for two broader outcome categories: mental distress (incl. general distress, depressive symptoms, anxiety symptoms, posttraumatic stress symptoms, substance use, and stress symptoms) and positive mental health (incl. life satisfaction, mental health-related quality of life, happiness, and well-being).

### Quality appraisal

Study quality was assessed by one senior team member (MS) using a modified version of AMSTAR 2 (*A Measurement Tool to Assess Systematic Reviews*; [41]). AMSTAR 2 is a critical appraisal tool for systematic reviews that include randomized and/or non-randomized studies of healthcare interventions. It assesses study quality using 16 items, covering areas such as registered study protocols, adequacy of literature search, and risk of bias. However, since AMSTAR 2 is only partially applicable to systematic reviews of non-intervention studies, we followed the recommendation of Puljak and colleagues [42] to use a subset of its items – specifically items 2, 4, 5, 6, 7, 10, 14, and 16.

To provide a better assessment of the quality of non-intervention reviews, we adapted this approach by incorporating 11 additional items from the PRISMA [43] checklist (1 for introduction, 4 for methods, 3 for results, 1 for discussion, and 1 for availability of data, code and other materials). This expanded approach aimed to account for critical elements such as the theoretical framework, methodology, results presentation, and derivation of implications. The final self-developed quality assessment tool (see Supplementary Material 3) consisted of 18 items. Based on the number of items that could be assessed per study, we calculated an overall study quality rating, ranging from 0% to 100%.

### Data synthesis

Based on the extracted data, a qualitative narrative synthesis of the included reviews was conducted in textual and tabular format, detailing the study population (e.g., descriptive statistics), resilience factors, mental health outcomes examined, and the societal challenges, stressors, and crises reported.

Resilience factors assessed at the social and societal levels were documented. For each factor, we assessed the frequency with which it has been studied and how many systematic reviews provide evidence for its association with mental health outcomes. The frequency of specific mental health outcomes (e.g., depressive symptoms, anxiety symptoms) mentioned in the included systematic reviews was also documented. For the purpose of this synthesis, mental health outcomes were summarized under the term “general mental health” due to the limited number of reviews available for analyzing the associations of specific mental health outcomes (e.g., depressive symptoms, anxiety symptoms, stress) with resilience factors. The frequency of each type of association was counted to identify the direction of the associations for each resilience factor. Factors with unfavorable effects were considered to be those resilience factors that increase the likelihood of greater mental distress and symptoms, while factors with favorable effects were associated with reduced mental distress and fewer symptoms. If fewer than 80% of the associations extracted from the systematic review clearly categorized a factor as a factor with favorable or unfavorable effects, the evidence was classified as mixed. If no significant associations were reported for a specific factor in the included reviews, these were categorized as non-significant associations.

### Implementation of the expert survey

Given the diverse perspectives on resilience factors and the limited attention some of those factors have received in resilience research, two expert interviews were conducted. One interview involved two experts in the field of family psychology to address social aspects and the proximal environment. The second interview was conducted with one public health expert and her research team, providing insights into societal factors and the more distal environment. These expert interviews followed an unstructured, open-ended discussion format, guided by the central question: “In your view, what are the key resilience or health-promoting factors when experiencing crises?”. The two interviews were documented, and key resilience and health-promoting factors were extracted based on the written interview protocols. With the help of these interviews, a comprehensive list of relevant social and societal resilience and health-promoting factors was compiled. The review team then evaluated whether these factors could be considered as potential resilience factors. This list was further expanded with factors identified from both the included and excluded reviews to generate a more exhaustive catalog of resilience factors for future research (see Supplementary Material 4). As a result, discrepancies emerged between the factors extracted from the included reviews (Research Question 4) and those identified in the expanded list (Research Question 5).

In the next phase, fifty selected international experts (and their research teams) were invited to participate in an online survey (hosted via www.soscisurvey.de). These experts were identified and selected based on their extensive publication records in the fields of resilience research, family psychology, and/or public health, as well as their contributions to key conceptual papers in those research areas. They were contacted via their public email address. The participation in the survey was completely anonymous, which also prohibited the collection of sociodemographic data. The survey was developed based on the created list of resilience factors. Experts were asked to rate the importance of each factor on a scale from 0 (*not at all important*) to 10 (*very important*). The survey was conducted between September 11 and October 31, 2023. Additionally, the 9th international Resilience Symposium in Mainz 2023 was used for participant recruitment. In this survey, participants were also invited to suggest additional relevant factors. The survey results were analyzed descriptively (see Supplementary Material 4), and in the final step, these findings were compared with the extracted factors from the systematic reviews to identify potential research gaps that merit further exploration.

The expert survey procedure was reviewed and approved as ethically acceptable by the Ethics Committee of the Universität des Saarlandes (Faculty of Empirical Human Sciences and Economics; ID: 25–38).

## Results

### Search outcomes

**Fig. 1:**
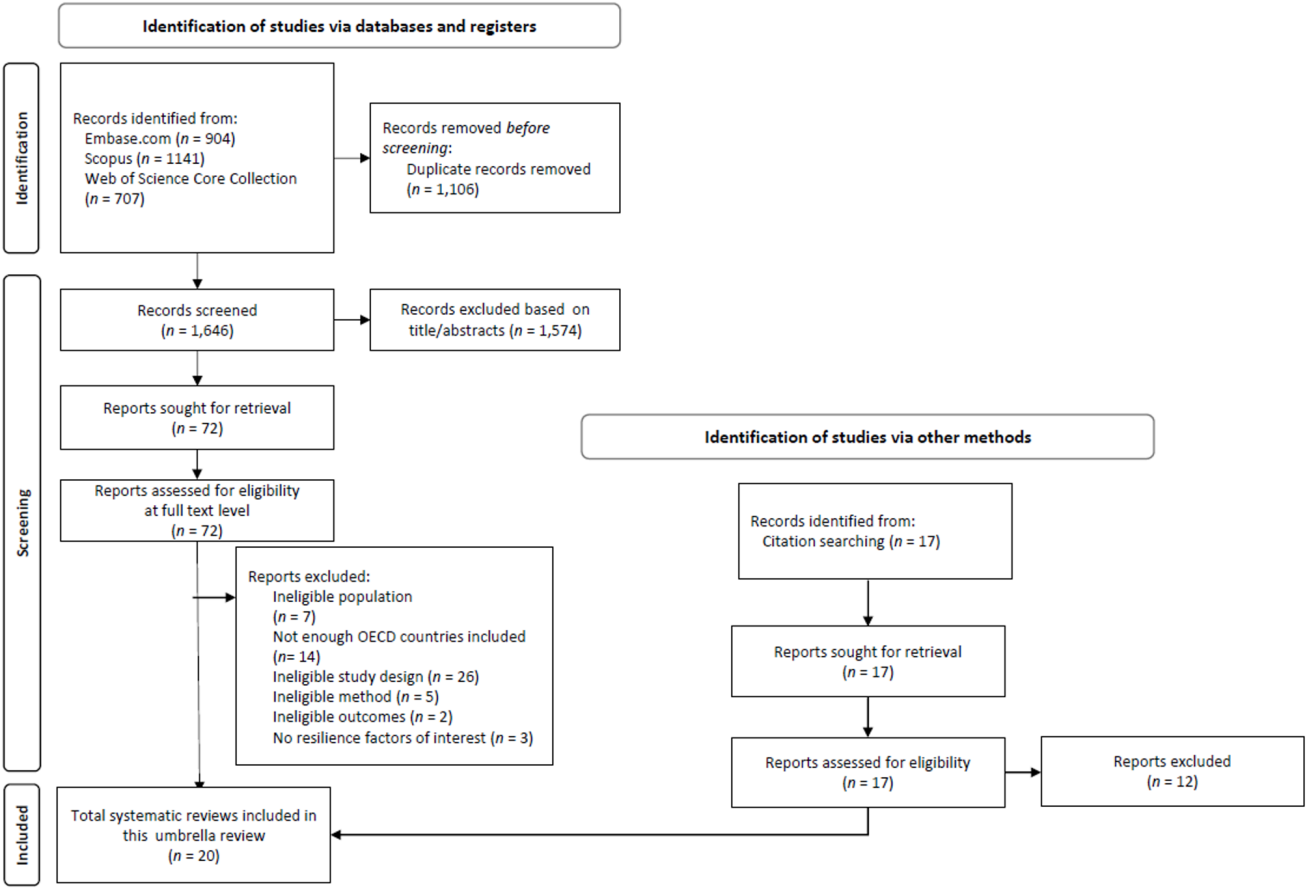
PRISMA flow diagram

The search for systematic reviews in electronic databases yielded 2,752 eligible records, from which 1,106 duplicates were removed (see PRISMA Flowchart in Fig. 1). Of the 1,646 records screened at the title/abstract level, 1,574 were excluded. This left 72 entries to be assessed for eligibility at the full-text level. Five additional records were obtained through citation searching of the included systematic reviews. In total, 20 eligible systematic reviews were included in this overview of systematic reviews (see Table 1). We systematically assessed the degree of overlap between the primary studies included in the systematic reviews using a Graphical Representation of Overlap for OVErviews (GROOVE) heatmap (see [64]). In total, we examined 190 pairs of reviews and found that in 189 cases, the overlap was slight (< 5%), with 186 of them showing 0% overlap. Only one case showed moderate overlap (5% to < 10%). The GROOVE heatmap is provided in Supplementary Material 5, and the underlying Excel file used to generate the heatmap has been uploaded to OSF.

**Table 1:** Characteristics of included systematic reviews

Study	Societal challenge	*N* (or range of *N*)	Population (age range, % female)	No. of included studies in systematic review	Mental health outcomes	Resilience factors examined	Extracted effect sizes	Overall quality assessment
Bekteshi 2020 [44]	Ethnic minority stress	23 − 10,001	Latino immigrants in the United States, NR, NR	30	Stress	*Social*: being married, household/family income, family conflict, received social support, family values *Societal*: acculturation, adherence to traditional gender roles	None	69.44%
Bogic 2015 [45]	Seeking refuge after war	16,010	War refugees, NR, NR	27	Anxiety symptoms, depressive symptoms, PTSD symptoms	*Social*: perceived social support, being married	None	83.33%
Bonati 2022 [46]	Pandemics (COVID-19 pandemic)	96–140,656	General population	105	Anxiety symptoms, depressive symptoms, general distress, well-being	*Social*: having a partner, cohabiting, living with family/others, household/family income	None	69.44%
Ciuffreda 2021 [47]	Pandemics (COVID-19 pandemic)	71 − 7,127	Older adults from general population, ≥ 60, 57.5%	11	Anxiety symptoms, depressive symptoms	*Social*: living with family/others, social media engagement, being married, relationship quality *Societal*: rural region	Living with family/others (ORs = 1.32, 2.79); social media engagement (ORs = 0.99, 1.05), being married (ORs = 0.665; 1.37), rural region (OR = 1.29) and depressive symptoms Social media engagement (ORs = 0.77, 0.81), rural region (OR = 1.20) and anxiety symptoms relationship quality (OR = 5.24) and depressive/anxiety symptoms	75.00%
Esposito 2021 [48]	Pandemics (COVID-19 pandemic)	75 − 4,260	General population, NR, NR	12	General distress	*Social*: received social support *Societal*: shared actions/goals, trust in institutions	None	66.67%
Fellmeth 2017 [49]	Migration	18,783	Pregnant or postpartum migrants from low-and middle-income countries, range mean ages: 24–33, 100%	45	Anxiety symptoms, depressive symptoms, PTSD symptoms	*Social*: being married, living with family/others, relationship quality, received social support, structural social support	Social support (OR = 0.3, *p* >.05; OR = 0.96, *p* <.05; OR = 0.34, *p* >. 05), being single (OR = 0.68, *p* >.05; OR = 1.73, *p >*.05; OR = 22.5, *p* <. 05), and depressive symptoms	86.11%
Glonti 2015 [50]	Economic crises	NR	General population, NR, NR	22	Depressive symptoms, general distress, happiness, stress	*Social*: being married, relationship quality, social/family relationship quality *Societal*: rural region	None	83.33%
Hajak 2021 [51]	Stress of asylum seekers and refugees	57 − 6,821	Asylum seekers/refugees/migrants, range: 12–76, range: 14.9%−60.0%	13	Anxiety symptoms, depressive symptoms, general distress, PTSD symptoms	*Social*: living with family/others, separation from family *Societal*: housing situation, integration in host country	None	58.33%
Hamwey 2020 [52]	Terrorist attacks (9/11 terrorist attacks)	52–71,437	General population, NR, NR	30	PTSD symptoms	*Social*: being married, perceived social support, social integration	None	58.33%
Inderbinen 2021 [53]	Gender identity minority stress	4,913	Transgender people, range of age means: 25.2–66.5, NR	14	Anxiety symptoms, depressive symptoms, suicidality	*Social*: community connectedness, received social support	None	52.78%
Jannesari 2020 [54]	Migration and asylum seeking	2,402	Asylum seekers, NR, 35.6%	21	General distress	*Social*: separation from family, family conflict	Separation from family (*r* =.02, *p* =.005) and general distress	75.00%
Ka’apu 2019 [55]	Ethnic minority stress (indigenous people)	33–252,989	General Population/adult indigenous people (Native American, Alaska Native, American Indian), 15–77, NR	38	Depressive symptoms, PTSD symptoms, substance use, suicidality	*Social*: being married, living with family/others, social/family relationship quality, perceived social support, family support, family involvement, received social support, family conflict, having a partner *Societal*: tradition/culture, low risk environments, availability of substances, historical loss, enculturation, community involvement, historical oppression, education and employment opportunities, rural region, rapid cultural change, remote areas	None	52.78%
Lieneck 2021 [56]	Pandemics (COVID-19 pandemic)	NR	General population and risk groups like nurses, NR, NR	42	Depressive symptoms, general distress, suicidality	*Social*: structural social support, family functioning, social media engagement, social isolation *Societal*: supportive social policies	None	69.44%
Lluch 2020 [57]	Pandemics (COVID-19 pandemic)	80 − 12,596	Healthcare personnel, NR, NR	76	Burnout symptoms, well-being	*Social*: perceived social support, being married, conflict setting at work, work safety measure *Societal*: distributive justice, procedural justice	Social support (*r* = −.508, *p* <.01), conflict setting at work (*r* =.426, *p* <.01) and burnout symptoms	52.78%
McCann 2018 [58]	Gender identity minority stress	10 − 1,229	Transgender people, NR, NR	21	Anxiety symptoms, depressive symptoms, general distress, substance use	*Social*: perceived social support, structural social support *Societal*: housing situation, supportive social policies, access to mental health services	None	61.11%
Reed 2021 [59]	Ethnic minority stress (African Americans)	NR	African Americans, NR, NR	20	Stress, suicidality, well-being	*Social*: social isolation, family support, received social support, church-based social support *Societal*: racial identity, role of women, lower occupational/income inequalities, religion	None	36.11%
Snijder 2021 [60]	Ethnic minority stress (Aboriginal and Torres Strait Islander Australians)	Range: 26–211,482	Aboriginal and Torres Strait Islander Australians, 5–80, NR	38	Substance use	*Social*: received social support, positive role models, relationship quality, being married, social engagement *Societal*: housing situation, substance availability, marginalization related to substance use, workplace promotes substance use, social/recreational opportunities in communities, access to mental health services, social opportunities in communities, rural region, remote areas, normalization of substance use, cultural obligation to share substances, cultural engagement, substance use policies	Social support (OR = 0.59), rural region (OR = 1.40), remote areas (ORs = 1.25; 1.54–1.58; 2.53), cultural engagement (ORs for males = 0.66; ORs for females = 0.76; 0.81) and substance use	83.33%
Vigny-Pau 2021 [61]	Gender identity minority stress	30 − 27,715	Transgender people, NR, NR	52	Suicidality	*Social*: perceived social support, family conflicts, family support *Societal*: rural region, supportive social policies, housing situation	None	66.67%
Ward 2013 [62]	Ethnic minority stress (African Americans)	8,833	African American men, age range: 18–65, 0%	19	Depressive symptoms, suicidality	*Social*: being married, living with family/others, structural social support, family support, household/family income *Societal*: job security	None	33.33%
Wesemann 2022 [63]	Terrorist attacks	159,621	Emergency responders, mean age range: 35.6–49, NR	33	General distress, PTSD symptoms	*Social*: social isolation, perceived social support, social integration *Societal*: access to mental health services	Social isolation and PTSD symptoms: OR = 8.4	72.22%

*NR* Not reported

### Study and sample characteristics

Table 1 presents the characteristics of the included systematic reviews published between 2013 and 2022. The primary studies included in those reviews were predominantly conducted in high-income OECD countries (at least over 66% of the primary studies included in the reviews).

Sample sizes across the systematic reviews ranged from 2,402 to 489,419 participants. Six reviews focused on adults from the general population, while 14 reviews examined specific high-risk populations (e.g., healthcare personnel, migrants, asylum seekers, war refugees, participants from ethnic, sexual, and gender minorities). Overall, many systematic reviews lacked detailed information on sample sizes, with data on age being reported even less frequently. Additionally, gender distributions were rarely mentioned, limiting the ability to draw comprehensive conclusions about the sociodemographic characteristics of included primary studies.

### Results of the quality appraisal

**Fig. 2:**
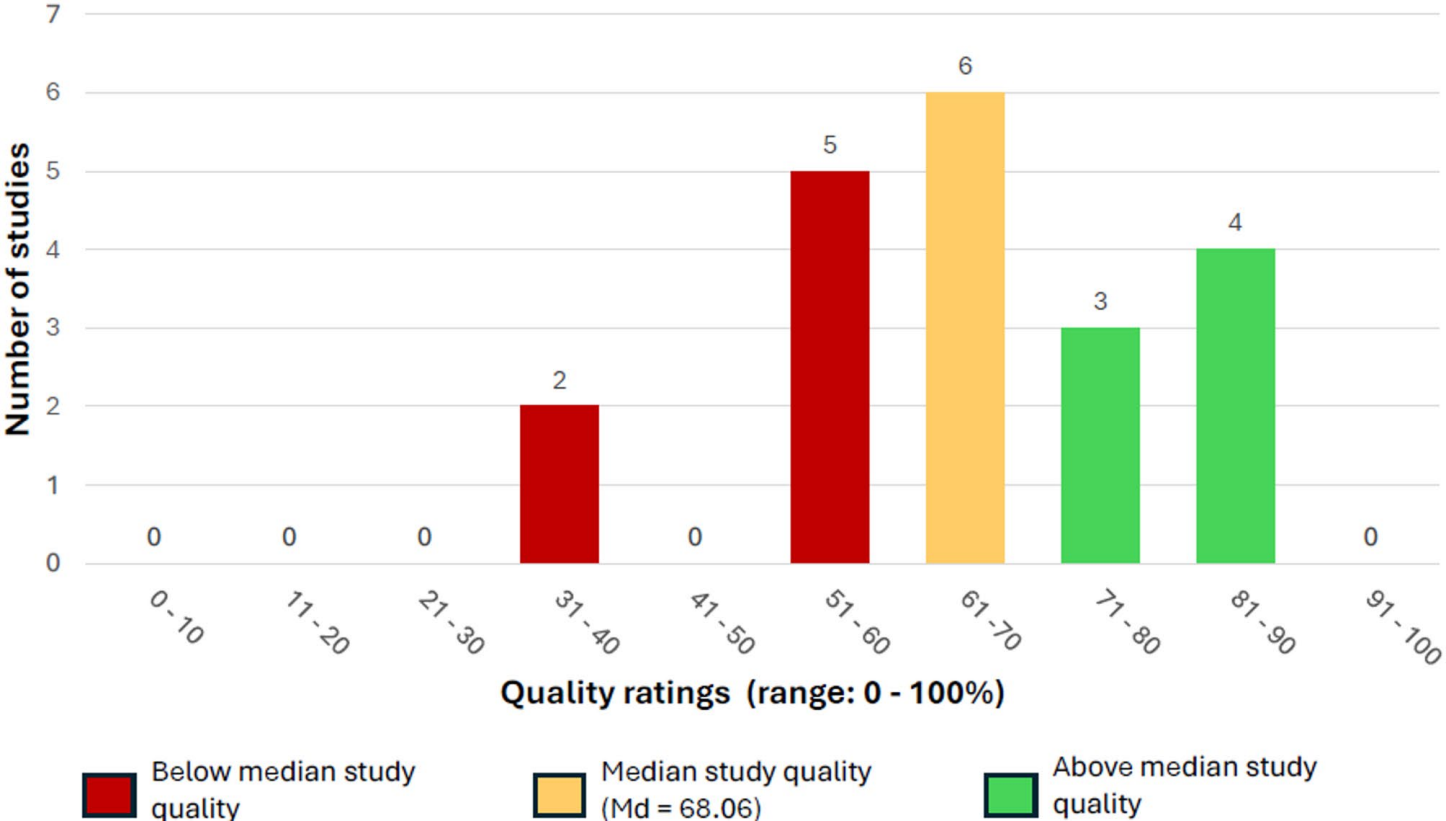
Distribution of study quality ratings

Overall, the quality of the included reviews was moderate (see Fig. 2), with a median quality rating of 68.06% (*M* = 65.28%, *SD* = 14.80; range: 33.33%–86.11%). The primary methodological shortcomings identified across the included reviews were as follows: none reported the sources of funding for the included primary studies (100.0% high risk), and most did not provide access to data, code, or other materials (95.0% high risk). Additionally, many reviews failed to include a discussion of heterogeneity (40.0% high risk). Furthermore, 65.0% of the reviews had not pre-registered their methods before the study’s conduct (high risk), and 35.0% did not assess the risk of bias of included primary studies.

#### Research question 1: which societal challenges and crises have been examined in the included systematic reviews?

Among the 20 systematic reviews included in this overview of systematic reviews, 5 reviews (25.0%) focused on pandemics (solely the COVID-19 pandemic), 5 reviews (25.0%) examined stress experienced by ethnic minorities, and 4 reviews (20.0%) explored issues related to migration and seeking asylum. Additionally, 3 reviews (15.0%) analyzed stress experienced by sexual and gender minorities, 2 reviews (10.0%) investigated the consequences of terrorist attacks, and 1 review (5.0%) reported on economic crises.

#### Research question 2: what types of mental health outcomes have been studied in relation to these challenges?

Depressive symptoms were examined most frequently (11 reviews, 55.0%), followed by general distress/general mental health examined in 8 reviews (40.0%) and anxiety symptoms assessed in 7 reviews (35.0%). PTSD symptoms and suicidality/suicide were each assessed as outcomes in 6 reviews (30.0%). Stress symptoms and substance use were examined in 3 reviews each (15.0%). The relationship between resilience factors and burnout was analyzed in a single review (5.0%). Well-being as a positive mental health outcome was examined in 3 reviews (15.0%), and happiness was assessed in one review (5.0%).

#### Research question 3: which social and societal resilience factors have been examined as correlates of these mental health outcomes?

All 20 systematic reviews examined social resilience factors, while only 13 reviews (65.0%) assessed societal resilience factors. Across the included reviews, 23 distinct social resilience factors and 35 societal resilience factors were identified (see Tables 2 and 3 for the complete list of resilience factors). Various forms of social support, such as *perceived/received support* and *structural social support*, were the most frequently studied social resilience factors, appearing in 15 reviews. This was followed by *being married*/*having a partner* (11 reviews) and *living with family or others* (5 reviews). In terms of societal resilience factors, the most evidence was available for *living in rural areas* compared to urban areas (5 reviews), the *housing situation* (4 reviews), and *supportive social policies* (3 reviews).

**Table 2:**
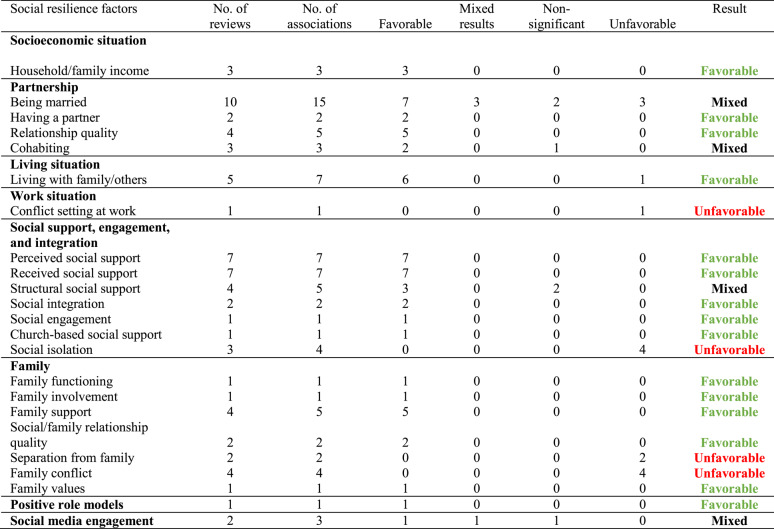
Overview of the social resilience factors, the number of systematic reviews in which they were analyzed, and extracted associations

Factors with unfavorable effects increase the likelihood of greater mental distress and symptoms, whereas factors with favorable effects are linked to reduced mental distress and fewer symptoms. If fewer than 80% of the extracted associations from reviews clearly categorized a factor as either a factor with favorable or unfavorable effects, the evidence was classified as mixed. When no significant associations were predominantly reported in the included reviews, these were categorized as non-significant associations

**Table 3:**
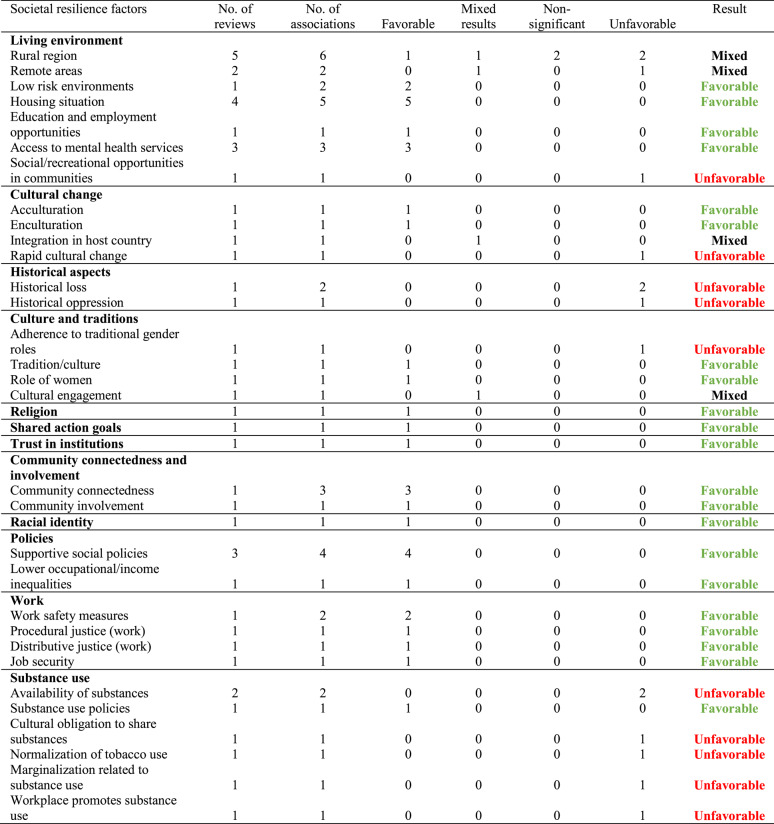
Overview of the societal resilience factors, the number of systematic reviews in which they were analyzed, and the extracted associations

Factors with unfavorable effects increase the likelihood of greater mental distress and symptoms, whereas factors with favorable effects are linked to reduced mental distress and fewer symptoms. If fewer than 80% of the extracted associations from reviews clearly categorized a factor as either a factor with favorable or unfavorable effects, the evidence was classified as mixed. When no significant associations were predominantly reported in the included reviews, these were categorized as non-significant associations

#### Research question 4: what associations between resilience factors and mental health outcomes have been observed?

Overall, 139 associations were extracted from the 20 included systematic reviews, encompassing 83 associations for social resilience factors and 56 for societal resilience factors. Of these, 93 associations (66.9%) indicated favorable links between resilience factors and mental health outcomes. Additionally, 30 associations demonstrated unfavorable associations (21.6%), while 8 (5.8%) showed mixed results, and 8 (5.8%) revealed non-significant associations between resilience factors and mental health outcomes.

In terms of *social resilience factors* (see Table 2), our overview of systematic reviews found that *household or family income* was examined in three reviews [44, 46, 62]. All three reviews demonstrated a favorable association between higher income and better mental health outcomes when facing societal challenges and crises.

In terms of partnerships, *having a partner* (two reviews: [46, 55]) and *higher relationship quality* (four reviews: [47, 49, 50, 60]) were consistently associated with better mental health outcomes, indicating a factor with favorable effects. The status of *being married* was frequently analyzed, yielding mixed results across various mental health outcomes. Four reviews identified marriage as a factor with favorable effects for seven outcomes [44, 50, 52, 55]. In contrast, three reviews reported mixed findings for three outcomes [45, 47, 49], while one review found no significant association with two mental health outcomes [45]. Additionally, three reviews found that marriage was also a factor with unfavorable effects for three mental health outcomes [50, 57, 62]. In the case of *cohabitation*, Bonati et al. [46] found that sharing everyday life with someone during the COVID-19 quarantine was associated with less general distress and cohabiting was also protective against suicide in the context of ethnic minority stress [55]. However, Fellmeth et al. [49] reported that cohabitation showed no association with depressive symptoms among migrants, leading to mixed results overall.

The associations between the social resilience factor of living *together with family or others* and several mental health outcomes was examined in four reviews, showing predominantly favorable associations with better mental health outcomes [46, 47, 51, 55]. However, the systematic review by Ward et al. [62] provided evidence that living in households with five or more people was linked to increased symptoms of depression when facing ethnic minority stress, indicating that living together with others may also act as a potential factor with unfavorable effects for worse mental health depending on the stressor context.

A *conflict* *setting at work* was examined only in a single review by Lluch et al. [57] and was found to be associated with higher burnout symptoms during the COVID-19 pandemic, thereby representing a factor with unfavorable effects.

Both *perceived social support* [45, 52, 55, 57, 58, 61, 63] and *received social support* [44, 48, 49, 53, 55, 59, 60] were examined in seven reviews each, with all reviews reporting favorable associations. This suggests that higher levels of social support are consistently associated with better mental health outcomes across various societal crises and challenges. Similarly, *social integration* [52, 63], *social engagement* [60], and *church-based social support* [59] were also identified as factors with favorable effects in the included systematic reviews. In contrast, *social isolation* was extracted from three reviews [56, 59, 63], revealing four associations that all demonstrated that increased isolation was linked to poorer mental health, identifying social isolation as a factor with unfavorable effects. For *structural social support* (i.e., social network size), the findings were mixed. While McCann et al. [58], Lieneck et al. [56] and Ward [62], identified a favorable association with better mental health, Fellmeth et al. [49] reported that anxiety and PTSD symptoms were not linked to the number of supportive people among migrants.

In the area of family dynamics, only favorable associations were found for *family functioning* [56], *family involvement* [55], *family support* [55, 59, 61, 62], *social/family relationship quality* [50, 55], and *family values* [44]. In contrast, *separation from the family* was identified as a factor with unfavorable effects in the reviews of Hajak et al. [51] and Jannesari et al. [54], which showed a connection to worse mental health. Furthermore, *family conflicts* were highlighted as a factor with unfavorable effects, with increased conflict within the family consistently linked to worse mental health in four reviews [44, 54, 55, 61].

Snijder et al. [60] demonstrated that *having positive role models*, who do not use tobacco, was identified as a factor with favorable effects against tobacco use when facing ethnic minority stress.

Regarding *social media engagement*, Ciuffreda et al. [47] found that contact with family and friends via social media was associated with both improvements and worsening of anxiety symptoms during the COVID-19 pandemic; however, having contact using social media was not significantly linked to depressive symptoms. In contrast, Lieneck et al. [56] reported that social media use among college-age students and young adults was associated with better mental health during the pandemic. The current evidence in this area can be characterized as mixed.

Most evidence regarding *societal resilience factors* (see Table 3) focused on the living environment. Concerning living in *rural regions*, one review reported a favorable effect [50], another found mixed results [60], one review found no significant association [47], and two reviews identified living in rural areas as a factor with unfavorable effects [55, 61]. This underscores the mixed and inconsistent evidence for this resilience factor.

Residing in *remote areas* was identified as a factor with unfavorable effects for increased suicidality [55], and mixed findings emerged for substance use [60], particularly in the context of experiencing ethnic minority stress.

Living in *low-risk environments* (e.g., environments with lower rates of poverty and violence) was identified as a factor with favorable effects in the systematic review by Ka’apu et al. [55] and associated with better mental health. A better *housing situation* (e.g., sufficient living space and enough housing options) was consistently shown to be a factor with favorable effects for five mental health outcomes across four reviews [51, 58, 60, 61]. Additionally, *education and employment opportunities* [55], as well as *access to mental health services* [58, 60, 63], were identified as factors with favorable effects in the systematic reviews. In contrast, *social and recreational opportunities within communities* were associated with increased substance use in the review by Snijder et al. [60], highlighting them as a potential factor with unfavorable effects.

In terms of cultural change processes, both *acculturation* [44] and *enculturation* [55] were identified as factors with favorable effects, each in single reviews. For better *integration into the host country*, mixed results were found in the review by Hajak et al. [51], while *rapid cultural change* was found to be a factor with unfavorable effects being associated with worse mental health in the review by Ka’apu et al. [55].

The identified historical aspects were both found to be factors with unfavorable effects in the review by Ka’apu et al. [55]. Specifically, *historical loss* (e.g., loss of culture, historical trauma, ethnic discrimination, poverty) was linked to increased substance use and suicidality, while *historical oppression* was associated with higher levels of PTSD symptoms.

*Tradition and culture* in general [55] and *the role of women in the community* (e.g., being courageous and having strength in family and community settings; [59]) were associated with better mental health in one review each. *Cultural engagement* showed mixed results concerning substance use [60], while *adherence to traditional gender roles* was identified as a factor with unfavorable effects for acculturative stress among Latino immigrants facing minority stress [44].

*Shared action goals* and *trust in institutions*, as reviewed by Esposito et al. [48], were associated with lower levels of general mental distress, establishing them as two potential social factors with favorable effects.

In terms of community factors, *community connectedness* emerged as a factor with favorable effects being associated with lower levels of depressive and anxiety symptoms, as well as reduced suicidality in transgender people facing minority stress [53]. Additionally, *community involvement* was identified as a factor with favorable effects against substance use among indigenous people facing minority stress [55].

The review by Reed et al. [59] demonstrated that *racial identity* was linked to psychological well-being and *religion* was an important factor with favorable effects against suicidality in African American men facing ethnic minority stress.

*Supportive social policies* (e.g., giving financial security or access to health services) were identified as a factor with favorable effects in three reviews [56, 58, 61], covering four mental health outcomes. Additionally, *lower occupational and income inequalities* were recognized as factors with favorable effects against stress in one review [59].

Regarding the workplace, *work safety measures* during the COVID-19 pandemic along with *procedural* (e.g., “My organization has a mechanism that allows employees to appeal decisions”) and *distributive* justice (e.g., “In general, the rewards I receive are fair”) were identified as factors with favorable effects associated with better mental health by Lluch et al. [57]. Additionally, the review by Ward et al. [62] found that *job security* was linked to fewer depressive symptoms in African American men facing ethnic minority stress.

The *availability of substances* was identified as a factor with unfavorable effects for substance use in the review by Snijder et al. [60] and for suicidality by Ka’apu et al. [55]. Only Snijder et al. [60] examined *substance use policies*, which were found to act as a factor with favorable effects. In the same review, *cultural obligations to share substances*,

*the normalization of tobacco use*, *marginalization related to substance use*, and *workplace environments that promote substance use* were identified as factors with unfavorable effects for increased substance consumption in the context of ethnic minority stress.

#### Research question 5: what social and societal factors do experts in the domains of resilience research, public health, and family studies consider to be important?

A total of *n* = 28 international experts in resilience research, family psychology, and public health participated in an online survey that assessed the importance of 72 resilience factors, comprising both 32 social and 40 societal factors. A comprehensive overview of all factors and their respective ratings is provided in Supplementary Material 4. Here, we highlight only the ten social and societal resilience factors rated as most important, along with the five factors rated as least important (Table 4).

**Table 4:** Ten social and societal resilience factors rated as most important, along with the five factors rated as least important by the experts participating in the online survey

**Social Resilience factors**			**Societal resilience factors**		
*10 most important resilience factors*					
	*M*	*SD*		*M*	*SD*
1. Overall social support	9.07	1.15	1. Financial safety and stability	8.61	1.20
2. Support from partners (e.g., emotional support)	8.93	0.92	2. Perceived physical safety	8.36	1.47
3. Relationship quality with a partner	8.74	1.20	3. Legal security/certainty	8.19	1.43
4. Social cohesion and connectedness (e.g., feeling a sense of belonging to a particular social group)	8.43	1.43	4. Job security	8.04	1.32
5. Family climate	8.38	1.06	5. Legal protection of minorities	7.96	1.48
6. Family acceptance (e.g., feeling of being accepted by family members)	8.33	1.52	6. Quality of workplaces and working conditions	7.79	1.50
7. Family functioning (e.g., how well family members get along)	8.26	1.56	7. Effective communication about crises	7.54	1.48
8. Social support from family members (e.g., parents, siblings)	8.08	1.26	8. Income equality	7.36	2.02
9. Higher family socioeconomic background	8.04	1.45	9. Social solidarity	7.28	1.49
10. Family cohesion	8.04	1.66	10. Trust in the legal system	7.22	2.08
*5 least important resilience factors*					
28. Social network size	4.32	1.95	36. Positive role models at a societal level (e.g., athletes, politicians)	5.08	1.72
29. Cross-cultural connections (e.g., having friends in other countries/cultures)	4.23	2.61	37. Collective religious and spiritual experiences	4.95	2.68
30. Number of persons living in a household (e.g., family, friends)	3.89	1.74	38. Living in rural areas	4.42	2.02
31. Number of family members	3.52	2.01	39. Living in urban areas	4.15	1.90
32. Number of colleagues at the workplace	2.63	1.90	40. Unavailability of alcohol (e.g., alcohol is only available from specific shops or for specific age groups)	4.08	2.75

Regarding social resilience factors, experts rated *overall social support* as the most important social resilience factor,followed by *emotional support from the partner* and *social cohesion/connectedness*. Next in importance were factors related to family dynamics (e.g., *family climate*, *family acceptance*, *family functioning*). The least important factors were simple measures of social contact, such as *social network size* and the *number of family members*.

*Financial safety and stability*, *perceived physical safety*, and *legal security and certainty* were rated as the most important societal resilience factors by the participating experts. In contrast, societal factors such as *positive role models at the societal level*, *residing in rural areas* or *urban areas*, and *the restricted availability of alcohol* were rated as the least important factors.

Additional suggested social factors by the experts include *teacher support* and the *provision of social support*. At the societal level, proposed factors encompass *freedom of speech and expression*, the *quality of available food in the area*, *societal openness to inclusion* (e.g., for individuals with mental health conditions, neurodiversity, or disabilities) as well as *racial and gender equity*.

## Discussion

This overview of systematic reviews aimed to examine social and societal resilience factors and their associations with mental health responses to societal challenges and crises in OECD member states. A total of 20 systematic reviews were identified, focusing on mental health outcomes in the context of various societal challenges, including pandemics, terrorist attacks, economic crises, and minority stress. According to these reviews, 58 resilience factors were examined, comprising 23 social resilience factors and 35 societal resilience factors. Social factors were rated as slightly more important for resilient outcomes than societal factors by the experts. However, for many resilience factors evidence is missing.

Depressive symptoms were the most frequently analyzed mental health outcome, followed by general distress/general mental health outcomes and anxiety symptoms. Notably, there has been a lack of research on positive mental health outcomes, with only 4 reviews examining positive mental health. Emerging research suggests that positive dimensions of mental health may independently influence both the onset and progression of mental health conditions [65]. Knowledge about resilience factors and their associations with positive mental health outcomes is needed. Accordingly, promoting well-being and cultivating positive mental health have gained increasing recognition as central objectives in public mental health strategies [66, 67]. Future research should aim to incorporate positive mental health outcomes such as subjective well-being or life satisfaction into their study designs.

While the number of social resilience factors was somewhat lower compared to the number of societal resilience factors, there was more evidence available regarding their associations with mental health outcomes. Overall, it can be noted that while certain social and societal resilience factors have more substantial evidence (e.g., *social support* or *housing situation*), many others are supported by only limited or isolated findings. This underscores the need for more primary studies and, in turn, systematic reviews that comprehensively examine both social and societal resilience factors in the face of societal challenges and crises. In addition, for some key factors, such as *being married* or *living in rural areas*, the evidence is very mixed. Further research is needed to identify the contextual factors that are likely to explain this heterogeneity.

The examination of the results from the expert survey indicates that, on average, social factors are rated as slightly more important than societal factors concerning their importance in achieving resilient outcomes following societal crises and challenges. According to the experts, social factors – particularly those related to the close, proximal social environment such as family and partnerships – play a more crucial role. This finding might also be influenced by the fact that resilience factors are typically defined in relation to mental health of individuals [16, 17]. As such, the emphasis is placed on what is important for the individual, rather than what is important for the community as a whole. Since social factors are more closely tied to the individual, they may have been rated as more important for individual mental health by the experts. Further qualitative studies and interviews are necessary to better understand the experts’ ratings.

Overall, different aspects of *social support* emerged as the most important factors with favorable effects, supported by robust evidence as a key social resilience factor. This finding is consistent in both the data obtained from the included systematic reviews and our expert survey. Social support remains one of the most extensively researched factors in resilience studies [27, 28, 68]. Future studies should further investigate the interplay between an individual’s need for social support, its sources, availability, and provision to better understand the role of social support during societal crises [69]. *Social isolation* also emerged as a factor with unfavorable effects with strong evidence linking it to poorer mental health, further underscoring the critical role of social support.

Additionally, it is evident that familial factors have been assigned particularly high importance by the experts. This aligns with the understanding that, during societal crises, the family often serves as the central social element to which individuals can turn for support [70–72]. Overall, the evidence from primary studies on familial factors is limited. However, potential factors with favorable effects with the most substantial evidence include *living with family/others* and *family support*. Having *family conflicts* appears to be a potential factor with unfavorable effects. Further research into family factors is urgently needed, not by the invention of entirely new concepts, but by building on and collaborating with the well-established field of family psychology and their concepts and constructs (e.g., family climate, family functioning, parenting style, family support). These aspects should be more often included in primary studies.

While *relationship quality* generally shows favorable associations, the findings regarding *marital status* are mixed. Current research typically considers only marital status (single vs. married vs. widowed), while relationship quality is rarely addressed. This approach is problematic, as an unhappy marriage or a marital separation can be a significant source of stress, while a happy marriage can show favorable associations with better mental health outcomes [73–75]. Similarly, the *quality of relationships* with family and friends has emerged as a factor with favorable effects, although the evidence for this is currently limited. Future studies should address this gap, as previous research has frequently focused on the number of supportive individuals rather than the quality of those relationships.

At the level of societal resilience factors, while there were many potential resilience factors identified, most showed only one or two associations with mental health outcomes, indicating that the evidence base is insufficient for most factors. Overall, there is strong evidence suggesting that a good *housing situation*, *access to mental health services*, and *supportive social policies* serve as potential factors with favorable effects. Additionally, while there was considerable evidence available for living in *rural versus urban regions*, the findings were mixed and more studies are needed. Interestingly, experts considered living in rural areas to be one of the least important societal resilience factors. 

*Financial safety*, *physical safety*, and *legal security* were identified by experts as the most important societal resilience factors; however, systematic reviews have yielded only very limited evidence concerning these factors. This highlights a significant research gap (e.g [76]), as assessing these aspects may pose challenges due to their nature as national-level factors and they could be difficult to quantify in primary studies being conducted in single countries. One option to assess these aspects could be to analyze them regionally (based on place of residence and municipal codes) and to link them with survey data.

Factors related to the *living environment*, such as the *availability of schools*,* physical facilities*, and the *quality of infrastructure*, were rated by experts as being of moderate to low importance. While there is somewhat more evidence regarding these aspects compared to other societal resilience factors, the overall amount of evidence remains insufficient.

No information could be extracted from included the systematic reviews for some important aspects, such as *social cohesion* (e.g., solidarity), which plays a critical role in recovery from crises like the COVID-19 pandemic [31]. It should also be noted that solidarity, in particular, was rated as an important societal resilience factor by the experts.

Overall, societal-level factors have been studied only sporadically in both this review and the existing literature. Therefore, future studies should place a stronger emphasis on the societal level and its role in fostering resilience during the recovery from societal crises [27, 30, 31, 33]. The provided results offer a good starting point.

### Strengths and limitations

One strength of our overview of systematic reviews is its focus on the role of social and societal resilience factors when facing societal challenges and crises, a research area that has received little attention in resilience research so far. By providing a comprehensive list of potential social and societal resilience factors, we have established an important evidence base for future studies in the field. This list has the potential to guide researchers in planning studies by identifying which factors should be assessed. Furthermore, the expert survey conducted as part of our study offers initial insights into the potential importance of these factors. Additionally, the tailored quality appraisal tool developed for this study is noteworthy, as it enabled a comprehensive assessment of the quality of the included systematic reviews.

Our findings must also be interpreted in light of several limitations. First, the scope of this overview of systematic reviews was restricted to systematic reviews to ensure a comprehensive and representative evidence base. However, this approach led to the exclusion of resilience factors for which only a limited number of primary studies currently exist.

Second, most of the findings are based on narrative syntheses performed in the included systematic reviews, which allowed for conclusions only about the direction of associations (i.e., favorable vs. unfavorable), without providing insights into the strength or causality of these relationships. This is due to the fact that many of the included systematic reviews did not report effect sizes or other statistical information (e.g., *p*-values) allowing for a more detailed synthesis. Therefore, quantitative synthesis of the data was not feasible. Furthermore, the total number of primary studies included in the systematic reviews could not be used as an indicator of the strength of evidence in our review as only a subset of the included systematic reviews specifically examined social and societal resilience factors. Consequently, only a smaller number of included primary studies per systematic review were actually relevant for our synthesis – not the total number of included studies. More complex study designs are needed to investigate the interactions and potential causal pathways between social and societal resilience factors and mental health outcomes [22, 77]. Furthermore, the methodological approach of this review precluded an assessment of how well the statistical models in primary studies were controlled for potential confounders. Data from models that include a larger number of control variables tend to diminish the explanatory power of individual predictors [78, 79]. In the related project based on a synthesis of primary studies, we observed that the inclusion of more variables and resilience factors in regression models was associated with less favorable evidence ratings for each resilience factor. Our results also indicated that resilience factors across all levels exhibited only small incremental validity beyond sociodemographic variables and other resilience- and health-promoting factors [27]. Future studies could incorporate factors from multiple levels (individual, social, and societal) simultaneously to better assess their incremental predictive value.

Third, the limited number of available systematic reviews and the small number of associations identified prevented the analysis of differences between mental health outcomes (e.g., differences between depressive and anxiety symptoms). For 7 social and 29 societal resilience factors, only single reviews provided information on associations being relevant for this overview of systematic reviews. Moreover, comparisons between the individual outcomes were further complicated by the fact that many mental health outcomes were assessed using different instruments – and in some cases, different versions of the same instrument (e.g., shortened item sets). Consequently, some heterogeneity in our findings may also result from between-study differences in outcome assessment.

Fourth, the selection of international experts in the fields of resilience research, family psychology, and public health for the expert survey may have introduced bias in assessing the importance of certain resilience factors. This could explain why, for instance, family-related factors were rated as highly important social resilience factors.

Fifth, many of the associations classified as *unfavorable* or *favorable* are based on a limited number of reviews and associations. Conclusions derived from a single review or a limited number of reviews should be interpreted with caution. To still allow for an indication of the directional trend of associations, we applied a strict criterion requiring that at least 80% of associations point in the same direction for a resilience factor to be labelled as either *unfavorable* or *favorable*. If this threshold was not met, the label *mixed* was assigned. We also aimed to be as transparent as possible regarding the number of reviews and associations on which the labelling was based (see Tables 2 and 3). However, receiving such a label does not imply that the direction of the association for a given resilience factor is well evidence-based. Further evidence is clearly needed to substantiate any such conclusion.

Sixth, the generalizability of the findings from this overview of reviews is subject to notable limitations. The limited number of existing systematic reviews has primarily focused on a narrow range of societal stressors (mainly the COVID-19 pandemic, ethnic and gender minority stress). Future research should investigate whether our findings apply to other societal stressors within the same broader category (e.g [14])., and explore whether specific social and societal resilience factors are uniquely beneficial for particular types of societal challenges and crises. Furthermore, it cannot be assumed that the resilience factors identified as important resources are equally relevant for all individuals within OECD member states exposed to a given stressor. Such a variation across cultures was recently found for the individual-level resilience factor *emotion regulation* [80]. For example, reappraisal tends to be more adaptive in cultures that are short-term oriented, uncertainty-tolerant, and competition-driven. Conversely, suppression was more maladaptive in indulgent, individualistic, and competition-driven cultures. The ability to read and interpret contextual cues that code demands and opportunities of a situation and enable the selection of appropriate coping strategies (context sensitivity), varies between individuals but is also shaped by cultural aspects [81]. Further studies are required to determine whether these resilience factors are universally applicable across diverse contexts. However, based on the previously cited literature, this appears unlikely. Furthermore, in the included systematic reviews the associations between resilience factors and mental health outcomes were not reported separately for cross-sectional and longitudinal primary studies. Moreover, some of the included systematic reviews did not consistently specify whether the underlying primary studies employed cross-sectional or longitudinal designs. Additionally, most reviews conducted narrative syntheses that combined findings from various study designs to draw overall conclusions. Consequently, cross-sectional and longitudinal studies were in some cases pooled for the interpretations of the included reviews.

### Implications for future research

Our overview of systematic reviews aimed to provide insights and guidance for future research on resilience in the context of societal crises and challenges. It highlights the need for more primary studies and systematic reviews that investigate both social and societal resilience factors, as these are critical for a comprehensive understanding of resilience responses to societal stressors. We have compiled a detailed list of potential resilience factors (see Supplementary Material 4), which can serve as an important resource for future research. Studies that examine a broad range of psychological resilience factors across different levels remain scarce [18, 82, 83]. A collaborative effort to define and regularly assess a core set of the most promising resilience factors at different levels could prove beneficial, enabling comparability of resilience factors across nations and cultures. Furthermore, this approach would help to explore cross-cultural differences, which have been largely overlooked. The list could also help to ensure that relevant social and societal resilience factors are systematically included in future studies [27].

The expert survey results highlight the potentially crucial role of families in achieving resilient outcomes when facing societal challenges and crises. Family-related resilience factors are rarely considered in resilience research, representing a notable gap in the existing literature [70–72].

The work environment also offers potential for further exploration. Given the significant amount of time people spend at work, the role of support from colleagues and supervisors remains highly underexplored in the context of societal challenges and crises (e.g [84, 85]).,.

Regarding societal resilience factors, most studies have concentrated on immediate environmental factors, such as urban versus rural settings or the availability of essential facilities like schools and mental health centers. However, resilience factors at the national level have been less frequently examined (e.g., financial safety and stability, or physical safety). Future research requires multinational studies to enable comparisons of these resilience factors at the national level and to enable the linkage of self-reported and regional/structural data.

Due to the large number of resilience factors across different levels, recent conceptual frameworks propose that a smaller set of *resilience mechanisms* (e.g., regulatory flexibility) might mediate the relationship between resilience factors and resilient outcomes [1, 2, 19]. Kalisch et al. [2] define resilience mechanisms as mental, physical, or behavioral processes (e.g., seeking help) that are activated during acute stress. Resilience factors, such as willingness to seek help or communication skills, are predispositions or conditions that increase the likelihood of activating these mechanisms and facilitate optimal coping with stressors. However, research on resilience mechanisms remains relatively underexplored (e.g [2, 86, 87]). In particular, there is a significant evidence gap concerning social and societal resilience mechanisms. Given the critical role that social support plays in fostering resilient outcomes, it is essential to investigate mechanisms that promote the establishment and maintenance of social relationships, which contribute to an overall sense of connectedness [88]. Other resilience mechanisms may also exist at the social and societal level, such as capacities for transformation and adaptation, decision-making, and community actions (e.g [31, 34, 89]). However, it remains unclear which social and societal resilience factors increase the likelihood of these processes occurring [89].

Understanding the dynamics of resilience factors over time is also essential. For instance, a recent study found that the importance of perceived social support fluctuated during the first year following exposure to a stressor, with its relevance varying based on the source of support [77]. This highlights the need for longitudinal research to explore how the significance of social and societal resilience factors evolves as individuals navigate different stages of recovery or adaptation after stressor exposure.

## Conclusion

The aim of this overview of systematic reviews was to identify the social and societal resilience factors examined in response to societal challenges and crises, and to summarize the evidence on their associations with mental health outcomes. There was evidence for the importance of both social and societal resilience factors, with several key factors being identified. At the social level, the strongest evidence emerged for the favorable effects of *social support*, *relationship quality*, *absence of family conflict*, and *family support*. At the societal level, factors such as *adequate housing conditions*, *access to mental health services*, and *supportive social policies* were linked to resilient outcomes. However, for most factors, evidence remained limited and/or was mixed including favorable, non-significant and/or unfavorable effects. The expert survey revealed that while certain potential social and societal resilience factors, deemed highly important by experts from various research fields (e.g., family psychology, public health), have received some research attention (e.g., social support, relationship quality, family support) evidence remains limited for many others. It should be noted that not all participating experts conduct research specifically focused on resilience. We deliberately included experts with diverse research backgrounds to broaden the perspective beyond the current evidence base for social and societal resilience factors, as it remains a relatively small research field, while a larger number of researchers study successful adaptation processes, matching the state-of-the-art definition of resilience. Thus, given their expertise in examining psychological adjustment processes during challenges and crises, these experts were able to provide valuable insights into potentially relevant social and societal resilience factors moving beyond the borders of resilience research.

Resilience research could benefit from broadening its scope to include aspects of families, partnerships, workplaces, and societal factors as critical components for resilient individual adaptation processes. To achieve this, appropriate modeling approaches and study designs are necessary. Future large-scale international studies on public mental health should aim to investigate a more comprehensive set of resilience factors within integrated models, with greater attention given to both social and societal resilience mechanisms. A core set of the most promising resilience factors across different levels needs to be identified.

## Supplementary Information

Supplementary Material 1.

## Data Availability

The datasets generated and/or analysed during the current study are available in the Open Science Framework (OSF) repository: (https:/osf.io/f4zty). Code for analysis is available at the Open Science Framework (OSF): (https:/osf.io/f4zty).
