# Supplementary material for: Current evidence and future directions for social and societal resilience factors in response to societal challenges and crises: an overview of systematic reviews and expert rating
Source: BMC Public Health. 2025 Nov 6;25:3816. doi: 10.1186/s12889-025-25285-5 (PMC12590817; doi:10.1186/s12889-025-25285-5)
Supplement: Supplementary file 1 — Supplementary Material 1. [file 12889_2025_25285_MOESM1_ESM.docx]

**Supplementary Material**

Related to “Current Evidence and Future Directions for Social and Societal Resilience Factors in Response to Societal Challenges and Crises: An Overview of Systematic Reviews and Expert Rating”

Max Supke^1,2^, Lea M. Schaubruch^1^, Caroline Cohrdes^3^, Corinna Kausmann^3^, & Sarah K. Schäfer^1,2*^ / Klaus Lieb^1,4*^

* Both authors contributed equally.

^1^ Leibniz Institute for Resilience Research, Mainz, Germany

^2^ Department of Clinical Psychology, Psychotherapy and Psychodiagnostics, Technische

Universität Braunschweig, Braunschweig, Germany

^3^ Department of Epidemiology and Health Monitoring, Robert Koch Institute, Berlin, Germany

^4^ Department of Psychiatry and Psychotherapy, University Medical Center of Johannes Gutenberg University, Mainz, Germany

Correspondence concerning this article should be addressed to Dr. Max Supke, Leibniz Institute for Resilience Research, Wallstraße 7, 55122 Mainz, Email: max.supke@lir-mainz.de

**Table of Contents**

Supplementary Material 1 – Checklist for Umbrella Reviews 2

[Supplementary Material 2 – Search Strategies per Database 4](#_Toc180417133)

[Supplementary Material 3 – Quality Appraisal Items 6](#_Toc180417134)

[Supplementary Material 4 – Detailed Results of the Expert Survey 13](#_Toc180417183)

Supplementary Material 5 – Overlap of Included Primary Studies in the Systematic
Reviews 17

# **Supplementary Material 1 –** **Checklist for Umbrella Reviews**

| **Section/topic** | **#** | **Checklist item** | **Location** |
| --- | --- | --- | --- |
| *Title and Abstract* |  |  |  |
| Title | 1 | Identify the reports as an overview of (systematic) reviews, an umbrella review, or a meta-epidemiologic study | Title Page |
| Structured summary | 2 | Provide a structured abstract | Abstract |
| *Introduction* |  |  |  |
| Rationale | 3 | Specify the rationale for the overview of reviews in the context of an already-formed body of knowledge on the topic | Introduction |
| Objectives | 4 | Describe a precise statement of questions | Objective |
| *Methods* |  |  |  |
| Protocol and registration | 5 | Report if an overview protocol was developed and if and where it can be obtained and provide registration information | Protocol and Pre-Registration |
| Criteria for considering reviews for the overview | 6 | Describe review characteristics and report characteristics for eligibility criteria | Inclusion Criteria |
| Information sources | 7 | State all information sources in the search and date least searched | Search Strategy |
| Search strategy | 8 | Specify full electronic search strategy including any limits used, such as language restriction | Search Strategy and Supplementary Material 2 |
| Review selection | 9 | Provide the process for selecting reviews and its relevant details | Study Selection |
| Additional searches to identify other relevant primary studies | 10 | Report whether and why additional searches were conducted to identify other eligible primary studies | Search Strategy and Figure 1 |
| Data extraction and management | 11 | State the processes of data extraction from included reviews and their relevant details | Data Extraction |
| Data items | 12 | Specify all items overview authors sought (e.g., PICOS, method, results, funding source) | Data Extraction; Data Synthesis; Extraction sheet on OSF |
| Assessment of methodological quality of included reviews | 13 | Describe methods used for assessing methodological quality and quality of evidence and how this information was used for analyses | Quality Appraisal |
| Data synthesis | 14 | Specify the methods of handling data and their details | Data Synthesis |
| *Results* |  |  |  |
| Review selection | 15 | Provide the details of review selection or a flow diagram of the overview process | Search Outcomes and Figure 1 |
| Review characteristics | 16 | Describe characteristics of each review (e.g., title, PICOS, number of studies and participants included, assessment of methodological quality of reviews, results of individual reviews) | Table 1 |
| Assessment of methodological quality of included reviews | 17 | Report the results of assessment of methodological quality and quality of evidence of each included review | Results of the Quality Appraisal |
| Synthesis of results | 18 | Summarize the main findings of the overview. If overview authors undertook data synthesis, present each summary measure with a confidence interval or a credible interval and measures of heterogeneity or inconsistency | Results: Research Questions 1-5 and Tables 2 and 3 |
| *Discussion* |  |  |  |
| Summary of evidence | 19 | Provide a concise summary of the main findings with the strength and shortcomings of evidence for each main outcome | Discussion |
| Limitations | 20 | Discuss limitations of the overview of review | Limitations |
| Conclusion | 21 | Present implications for practice and future research | Implication for Future Research and Conclusion |
| Funding | 22 | Describe source of funding for overview of reviews | Declarations |

Source**:** Onishi, A., & Furukawa, T. A. (2016). State-of-the-art reporting. In G. Biondi-Zoccai (Ed.), *Umbrella reviews* (pp. 189–202). Springer. <https://doi.org/10.1007/978-3-319-25655-9_13>^[[1]](#footnote-1)^

# **Supplementary Material 2 – Search Strategies per Database**

**Embase.com including PubMed and Medline**

| **#** | **Query** |
| --- | --- |
| 1 | 'social'/exp OR social OR societal OR 'family'/exp OR family OR 'community'/exp OR community OR 'public'/exp OR public:ti,ab,kw |
| 2 | 'resilience factor*' OR 'psychosocial resource*' OR 'health-promoting factor*' OR 'health-benefitting factor*' OR 'protective factor*':ti,ab,kw |
| 3 | #1 AND #2 |
| 4 | #3 AND (2013:py OR 2014:py OR 2015:py OR 2016:py OR 2017:py OR 2018:py OR 2019:py OR 2020:py OR 2021:py OR 2022:py OR 2023:py) AND 'Review'/it |

**Scopus.com**

| **#** | **Query** |
| --- | --- |
| 1 | TITLE-ABS-KEY ("resilience factor*" OR "psychosocial resource*" OR  "health-promoting factor*" OR "health-benefitting factor*" OR "protective  factor*") |
| 2 | TITLE-ABS-KEY (“social” OR “societal” OR “family” OR  “community” OR “public”) |
| 3 | #1 AND #2 (in 2013 - 2023) |
| **Full search string**: ( TITLE-ABS-KEY ( "resilience factor*" OR "psychosocial resource*" OR  "health-promoting factor*" OR "health-benefitting factor*" OR "protective  factor*" ) ) AND ( TITLE-ABS-KEY ( social OR societal OR family OR  community OR public ) ) AND ( LIMIT-TO ( PUBYEAR , 2023 ) OR LIMITTO  ( PUBYEAR , 2022 ) OR LIMIT-TO ( PUBYEAR , 2021 ) OR LIMITTO  ( PUBYEAR , 2020 ) OR LIMIT-TO ( PUBYEAR , 2019 ) OR LIMITTO  ( PUBYEAR , 2018 ) OR LIMIT-TO ( PUBYEAR , 2017 ) OR LIMITTO  ( PUBYEAR , 2016 ) OR LIMIT-TO ( PUBYEAR , 2015 ) OR LIMITTO  ( PUBYEAR , 2014 ) OR LIMIT-TO ( PUBYEAR , 2013 ) ) AND ( LIMITTO  ( DOCTYPE , "re" ) ) AND ( LIMIT-TO ( SUBJAREA , "MEDI" ) OR LIMITTO  ( SUBJAREA , "PSYC" ) OR LIMIT-TO ( SUBJAREA , "SOCI" ) OR LIMITTO  ( SUBJAREA , "NURS" ) OR LIMIT-TO ( SUBJAREA , "NEUR" ) OR LIMITTO  ( SUBJAREA , "ENVI" ) OR LIMIT-TO ( SUBJAREA , "ARTS" ) OR LIMITTO  ( SUBJAREA , "MULT" ) OR LIMIT-TO ( SUBJAREA , "HEAL" ) ) | |

**Web of Science**

| **#** | **Query** |
| --- | --- |
| 1 | TS=(“social” OR “societal” OR “family” OR “community” OR public”) |
| 2 | TS=("resilience factor*" OR "psychosocial resource*" OR "health-promoting factor*" OR "health-benefitting factor*" OR "protective factors*") |
| 3 | #2 AND #1 |
| 4 | #2 AND #1 and 2023 or 2021 or 2022 or 2020 or 2019 or 2018 or 2017 or 2016 or 2015 or 2014 or 2013 (Publication Years) |
| 5 | #2 AND #1 and 2023 or 2021 or 2022 or 2020 or 2019 or 2018 or 2017 or 2016 or 2015 or 2014 or 2013 (Publication Years) and Review Article (Document Types) |

| **Supplementary Material 3 – Quality Appraisal Items**  **Using items from AMSTAR2 and the PRISMA Checklist 2020** | | | |
| --- | --- | --- | --- |
| **Original items** | **Item description and modifications** | **Reasons for modifications** | **Response options** |
| **Items selected from A MeaSurement Tool to Assess systematic Reviews (AMSTAR2)** | | | |
| Item 2: Did the report of the review contain an explicit statement that the review methods were established prior to the conduct of the review and did the report justify any significant deviations from the protocol? | **For Yes**: As for partial yes, plus the protocol should be registered and should also have specified:   - a meta-analysis/synthesis plan, if appropriate, and - a plan for investigating causes of heterogeneity - justification for any deviations from the protocol   **For Partial Yes:** The authors state that they had a written protocol or guide that included ALL the following:   - review question(s) - a search strategy - inclusion/exclusion criteria - a risk of bias assessment   **Not fulfilled** | Not modified | 1  0,5  0 |
| Item 4: Did the review authors use a comprehensive literature search strategy? | **For Yes, should also have (all the following):**   - searched the reference lists / bibliographies of included studies - If applicable: searched trial/study registries - included/consulted content experts in the field - where relevant, searched for grey literature - conducted search within 24 months of completion of the review   **For Partial Yes (all the following):**   - searched at least 2 databases (relevant to research question) - provided key word and/or search strategy - justified publication restrictions (e.g. language)   **Not fulfilled** | If applicable: searched trial/study registries | 1  0,5  0 |
| Item 5: Did the review authors perform study selection in duplicate? | **For Yes**, **either ONE of the following:**   - at least two reviewers independently agreed on selection of eligible studies and achieved consensus on which studies to include - OR two reviewers selected a sample of eligible studies and achieved good agreement (at least 80 percent), with the remainder selected by one reviewer   **Not fulfilled** | Not modified | 1  0 |
| Item 6: Did the review authors perform data extraction in duplicate? | **For Yes, either ONE of the following:**   - at least two reviewers achieved consensus on which data to extract from included studies - OR two reviewers extracted data from a sample of eligible studies and achieved good agreement (at least 80 percent), with the remainder extracted by one reviewer.   **Not fulfilled** | Not modified | 1  0 |
| Item 7: Did the review authors provide a list of excluded studies and justify the exclusions? | **For Yes, must also have:**   - justified the exclusion from the review of each potentially relevant study   **For Partial Yes:**   - provided a list of all potentially relevant studies that were read in full-text form but excluded from the review   **Not fulfilled** | Not modified | 1  0,5  0 |
| Item 10: Did the review authors report on the sources of funding for the studies included in the review? | **For Yes**   - must have reported on the sources of funding for individual studies included in the review.   Note: Reporting that the reviewers looked for this information but it was not reported by study authors also qualifies  **Not fulfilled** | Not modified | 1  0 |
| Item 14: Did the review authors provide a satisfactory explanation for, and discussion of, any heterogeneity observed in the results of the review? | **For Yes:**   - there was no significant heterogeneity in the results - OR if heterogeneity was present the authors performed an investigation of sources of any heterogeneity in the results and discussed the impact of this on the results of the review   **Not fulfilled** | Not modified | 1  0 |
| Item 16: Did the review authors report any potential sources of conflict of interest, including any funding they received for conducting the review? | **For Yes:**   - the authors reported no competing interests OR - the authors described their funding sources and how they managed potential conflicts of interest   **Not fulfilled** | Not modified | 1  0 |
| **Items selected from the PRISMA 2020 Checklist** | | | |
| Introduction: Rationale and objective | **For Yes:**   - describe the rationale for the review in the context of existing knowledge - provide an explicit statement of the objective(s) or question(s) the review addresses.   **Not fulfilled** | Not modified | 1  0 |
| Methods: Eligibility criteria | **For Yes:**   - specify the inclusion and exclusion criteria for the review and how studies were grouped for the syntheses.   **Not fulfilled** | Not modified | 1  0 |
| Methods: Selection and data collection process | - **For Yes:** - specify the methods used to decide whether a study met the inclusion criteria of the review, including how many reviewers screened each record and each report retrieved, whether they worked independently, and if applicable, details of automation tools used in the process. - specify the methods used to collect data from reports, including how many reviewers collected data from each report, whether they worked independently, any processes for obtaining or confirming data from study investigators, and if applicable, details of automation tools used in the process   **Not fulfilled** | Not modified | 1  0 |
| Methods: Synthesis methods | **For Yes:**   - describe any methods used to synthesize results and provide a rationale for the choice(s). If meta-analysis was performed, describe the model(s), method(s) to identify the presence and extent of statistical heterogeneity, and software package(s) used.   **Not fulfilled** | Not modified | 1  0 |
| Methods: Risk of bias assessment | **For Yes:**   - specify the methods used to assess risk of bias in the included studies, including details of the tool(s) used, how many reviewers assessed each study and whether they worked independently, and if applicable, details of automation tools used in the process.   **Not fulfilled** | Not modified | 1  0 |
| Results: Results of individual studies | **For Yes:**   - for all outcomes, present, for each study: (a) summary statistics for each group (where appropriate) and (b) an effect estimate and its precision (e.g., confidence/credible interval), ideally using structured tables or plots**.**   **Not fulfilled** | Not modified | 1  0 |
| Results: Results of syntheses | **For Yes:**   - for each synthesis, briefly summarise the characteristics and risk of bias among contributing studies. - present results of all statistical syntheses conducted. If meta-analysis was done, present for each the summary estimate and its precision (e.g. confidence/credible interval) and measures of statistical heterogeneity. If comparing groups, describe the direction of the effect. - present results of all investigations of possible causes of heterogeneity among study results. - present results of all sensitivity analyses conducted to assess the robustness of the synthesized results   **Not fulfilled** | Not modified | 1  0 |
| Results: Risk of bias | **For Yes:**   - present assessments of risk of bias for each included study.   **Not fulfilled** | Not modified | 1  0 |
| Discussion: Interpretation, limitations, and implications | **For Yes:**   - provide a general interpretation of the results in the context of other evidence. - discuss any limitations of the evidence included in the review. - discuss any limitations of the review processes used. discuss implications of the results for practice, policy, and future research.   **Not fulfilled** | Not modified | 1  0 |
| Availability of data, code and other | **For Yes:**  Report which of the following are publicly available and where they can be found:   - template data collection forms; - data extracted from included studies; - data used for all analyses; - analytic code; - any other materials used in the review   **Not fulfilled** | Not modified | 1  0 |
| Total points |  |  | **Total: 18 points** |

# **Supplementary Material 4 – Detailed Results of the Expert Survey**

**Table SM3.**

*This Table Presents the Detailed Results of the Expert Survey (n = 28). Experts Were Asked to Rate the Importance of Social and Societal Resilience Factors on a Scale From 0 (Not at All Important) to 10 (Very Important). Additionally, They Could Indicate Whether a Higher Level of a Given Factor Represents a Risk Factor, Thereby Decreasing the Likelihood of Resilience Outcomes.*

| **Social resilience factors** | ***M*** | ***SD*** | **Range** | **Minimum** | **Maximum** | **Rather a risk factor** |
| --- | --- | --- | --- | --- | --- | --- |
| 1. Overall social support | 9.07 | 1.15 | 4 | 6 | 10 | 0 |
| 2. Support from partners (e.g., emotional support) | 8.93 | 0.92 | 4 | 6 | 10 | 0 |
| 3. Relationship quality with a partner | 8.74 | 1.20 | 4 | 6 | 10 | 0 |
| 4. Social cohesion and connectedness (e.g., feeling a sense of belonging to a particular social group) | 8.43 | 1.43 | 7 | 3 | 10 | 0 |
| 5. Family climate | 8.38 | 1.06 | 3 | 7 | 10 | 0 |
| 6. Family acceptance (e.g., feeling of being accepted by family members) | 8.33 | 1.52 | 7 | 3 | 10 | 0 |
| 7. Family functioning (e.g., how well family members get along) | 8.26 | 1.56 | 8 | 2 | 10 | 0 |
| 8. Social support from family members (e.g., parents, siblings) | 8.08 | 1.26 | 6 | 4 | 10 | 0 |
| 9. Higher family socioeconomic background | 8.04 | 1.45 | 5 | 5 | 10 | 0 |
| 10. Family cohesion | 8.04 | 1.66 | 8 | 2 | 10 | 0 |
| 11. Social participation (e.g., activities that allow people to spend time and connect with others) | 7.68 | 1.87 | 7 | 3 | 10 | 0 |
| 12. Appreciation by social network (e.g., family, friends) | 7.67 | 1.34 | 5 | 5 | 10 | 0 |
| 13. Family adaptability (e.g., ability to flexibly adapt to changes and challenges) | 7.65 | 2.06 | 8 | 2 | 10 | 0 |
| 14. Positive social role models in the family | 7.60 | 1.73 | 8 | 2 | 10 | 0 |
| 15. Work-life balance | 7.39 | 1.77 | 7 | 3 | 10 | 0 |
| 16. Support from leadership at work | 7.31 | 1.32 | 5 | 5 | 10 | 0 |
| 17. Type of communication in the family | 7.28 | 2.11 | 8 | 1 | 9 | 0 |
| 18. Type of conflict resolution | 7.12 | 1.97 | 8 | 2 | 10 | 0 |
| 19. Support from colleagues at work | 7.12 | 1.37 | 7 | 3 | 10 | 0 |
| 20. Parenting style | 7.09 | 2.56 | 10 | 0 | 10 | 0 |
| 21. Quality of co-parenting | 7.08 | 2.17 | 8 | 2 | 10 | 1 |
| 22. Community support | 6.67 | 1.64 | 7 | 2 | 9 | 0 |
| 23. Religious support or spiritual support (e.g., a feeling of support in a religious/spiritual community) | 6.09 | 2.35 | 9 | 1 | 10 | 0 |
| 24. Being in a romantic relationship | 5.75 | 2.15 | 8 | 1 | 9 | 0 |
| 25. Social support from neighbors | 5.40 | 2.52 | 10 | 0 | 10 | 0 |
| 26. Digital social connectedness (e.g., feeling a sense of belonging via social media) | 5.30 | 1.74 | 6 | 2 | 8 | 4 |
| 27. Family religiousness or spirituality (e.g., participation in regular meetings) | 4.60 | 2.19 | 7 | 1 | 8 | 1 |
| 28. Social network size | 4.32 | 1.95 | 7 | 1 | 8 | 0 |
| 29. Cross-cultural connections (e.g., having friends in other countries/cultures) | 4.23 | 2.61 | 8 | 0 | 8 | 0 |
| 30. Number of persons living in a household (e.g., family, friends) | 3.89 | 1.74 | 6 | 1 | 7 | 0 |
| 31. Number of family members | 3.52 | 2.01 | 9 | 0 | 9 | 0 |
| 32. Number of colleagues at the workplace | 2.63 | 1.90 | 7 | 0 | 7 | 0 |
|  |  |  |  |  |  |  |
| **Societal resilience factors** |  |  |  |  |  |  |
| 1. Financial safety and stability | 8.61 | 1.20 | 5 | 5 | 10 | 0 |
| 2. Perceived physical safety | 8.36 | 1.47 | 5 | 5 | 10 | 0 |
| 3. Legal security/certainty | 8.19 | 1.43 | 6 | 4 | 10 | 0 |
| 4. Job security | 8.04 | 1.32 | 5 | 5 | 10 | 0 |
| 5. Legal protection of minorities | 7.96 | 1.48 | 6 | 4 | 10 | 0 |
| 6. Quality of workplaces and working conditions | 7.79 | 1.50 | 5 | 5 | 10 | 0 |
| 7. Effective communication about crises | 7.54 | 1.48 | 6 | 4 | 10 | 0 |
| 8. Income equality | 7.36 | 2.02 | 7 | 3 | 10 | 1 |
| 9. Social solidarity | 7.28 | 1.49 | 6 | 4 | 10 | 1 |
| 10. Trust in the legal system | 7.22 | 2.08 | 8 | 2 | 10 | 0 |
| 11. Availability of health care in the neighborhood | 7.15 | 2.27 | 9 | 1 | 10 | 0 |
| 12. Availability of cultural and social facilities (e.g., theatre, cinemas, youth clubs) | 7.11 | 1.28 | 6 | 3 | 9 | 0 |
| 13. Social acceptance at a societal level | 7.11 | 1.81 | 8 | 2 | 10 | 0 |
| 14. Collective identity or sense of community (e.g., feeling a sense of belonging and connectedness to the broader society) | 7.08 | 1.60 | 6 | 4 | 10 | 1 |
| 15. Trust in public institutions | 7.07 | 1.88 | 7 | 3 | 10 | 0 |
| 16. Greenness (e.g., access to natural green spaces such as parks) | 6.93 | 1.90 | 8 | 2 | 10 | 0 |
| 17. House affordability | 6.93 | 2.04 | 8 | 2 | 10 | 0 |
| 18. Trust in the political systems | 6.92 | 1.81 | 7 | 3 | 10 | 1 |
| 19. Integration of migrants at a national level | 6.84 | 2.27 | 8 | 2 | 10 | 0 |
| 20. Availability of schools in the neighborhood | 6.81 | 1.92 | 7 | 3 | 10 | 0 |
| 21. Average income | 6.71 | 1.82 | 7 | 3 | 10 | 0 |
| 22. Being involved in the community | 6.67 | 1.79 | 7 | 2 | 9 | 0 |
| 23. Availability of physical facilities in the neighborhood (e.g., sport facilities, health facilities) | 6.63 | 2.19 | 8 | 2 | 10 | 0 |
| 24. Perceived citizen efficacy (e.g., the perception that civil actions and decisions can result in societal change) | 6.56 | 1.99 | 7 | 2 | 9 | 0 |
| 25. Neighborhood income | 6.50 | 2.30 | 8 | 2 | 10 | 0 |
| 26. Access to mobile phones and internet | 6.46 | 1.84 | 8 | 1 | 9 | 1 |
| 27. Openness of societies towards other cultures and other forms of living | 6.41 | 2.65 | 10 | 0 | 10 | 0 |
| 28. Quality of infrastructure (e.g., public transport, walkability, bike lanes) | 6.26 | 1.89 | 7 | 2 | 9 | 0 |
| 29. Pollution (e.g., air, noise, toxin) | 6.17 | 2.92 | 9 | 1 | 10 | 15 |
| 30. Availability of stores in the neighborhood | 6.15 | 1.78 | 7 | 2 | 9 | 0 |
| 31. Cultural attachment and cultural connectedness (e.g., the extent to which an individual identifies with and is engaged in their culture) | 5.96 | 2.19 | 7 | 2 | 9 | 0 |
| 32. Blueness (e.g., access to natural blue spaces such as lakes) | 5.93 | 1.96 | 8 | 1 | 9 | 0 |
| 33. Political participation | 5.72 | 2.49 | 9 | 0 | 9 | 1 |
| 34. Size of home | 5.67 | 1.90 | 8 | 2 | 10 | 0 |
| 35. Societal recognition as a member of the workforce (e.g., healthcare staff during the pandemic) | 5.56 | 2.58 | 9 | 0 | 9 | 1 |
| 36. Positive role models at a societal level (e.g., athletes, politicians) | 5.08 | 1.72 | 7 | 2 | 9 | 0 |
| 37. Collective religious and spiritual experiences | 4.95 | 2.68 | 9 | 0 | 9 | 1 |
| 38. Living in rural areas | 4.42 | 2.02 | 8 | 0 | 8 | 1 |
| 39. Living in urban areas | 4.15 | 1.90 | 7 | 0 | 7 | 6 |
| 40. Unavailability of alcohol (e.g., alcohol is only available from specific shops or for specific age groups) | 4.08 | 2.75 | 10 | 0 | 10 | 0 |

|  | Bekteshi_2020 | Bogic_2015 | Bonati_2022 | Ciuffreda_2021 | Esposito_2021 | Fellmeth_2017 | Glonti_2015 | Hajak_2021 | Hamwey_2020 |  |  |  |  |  |  |  |  |  |  |
| --- | --- | --- | --- | --- | --- | --- | --- | --- | --- | --- | --- | --- | --- | --- | --- | --- | --- | --- | --- |
|  |  |  |  |  |  |  |  |  |  | **190** | **=** | **Total nodes (pairs of reviews)** | | | | |  |  |  |
| Bogic_2015 | 0,0% |  |  |  |  |  |  |  |  | **189** | = | Slight overlap (<5%) | | | | |  |  |  |
| Bonati_2022 | 0,0% | 0,0% |  |  |  |  |  |  |  | **1** | = | Moderate overlap (5% to <10%) | | | | |  |  |  |
| Ciuffreda_2021 | 0,0% | 0,0% | 0,0% |  |  |  |  |  |  | **0** | = | High overlap (10% to <15%) | | | | |  |  |  |
| Esposito_2021 | 0,0% | 0,0% | 0,0% | 0,0% |  |  |  |  |  | **0** | = | Very High overlap (>15%) | | | | |  |  |  |
| Fellmeth_2017 | 0,0% | 0,0% | 0,0% | 0,0% | 0,0% |  |  |  |  | Inderbinen_2021 | Jannesari_2020 | Ka'apu_2019 | Lieneck_2021 | Lluch_2022 | McCann_2018 | Reed_2021 | Snijder_2021 | Vigni-Pau_2021 | Ward_2013 |
| Glonti_2015 | 0,0% | 0,0% | 0,0% | 0,0% | 0,0% | 0,0% |  |  |  |  |  |  |  |  |  |  |  |  |  |
| Hajak_2021 | 0,0% | 0,0% | 0,0% | 0,0% | 0,0% | 0,0% | 0,0% |  |  |  |  |  |  |  |  |  |  |  |  |
| Hamwey_2020 | 0,0% | 0,0% | 0,0% | 0,0% | 0,0% | 0,0% | 0,0% | 0,0% |  |  |  |  |  |  |  |  |  |  |  |
| Inderbinen_2021 | 0,0% | 0,0% | 0,0% | 0,0% | 0,0% | 0,0% | 0,0% | 0,0% | 0,0% |  |  |  |  |  |  |  |  |  |  |
| Jannesari_2020 | 0,0% | 0,0% | 0,0% | 0,0% | 0,0% | 0,0% | 0,0% | 0,0% | 0,0% | 0,0% |  |  |  |  |  |  |  |  |  |
| Ka'apu_2019 | 0,0% | 0,0% | 0,0% | 0,0% | 0,0% | 0,0% | 0,0% | 0,0% | 0,0% | 0,0% | 0,0% |  |  |  |  |  |  |  |  |
| Lieneck_2021 | 0,0% | 0,0% | 0,0% | 0,0% | 0,0% | 0,0% | 0,0% | 0,0% | 0,0% | 0,0% | 0,0% | 0,0% |  |  |  |  |  |  |  |
| Lluch_2022 | 0,0% | 0,0% | 0,0% | 0,0% | 0,0% | 0,0% | 0,0% | 0,0% | 0,0% | 0,0% | 0,0% | 0,0% | 0,0% |  |  |  |  |  |  |
| McCann_2018 | 0,0% | 0,0% | 0,0% | 0,0% | 0,0% | 0,0% | 0,0% | 0,0% | 0,0% | 5,9% | 0,0% | 0,0% | 0,0% | 0,0% |  |  |  |  |  |
| Reed_2021 | 0,0% | 0,0% | 0,0% | 0,0% | 0,0% | 0,0% | 0,0% | 0,0% | 0,0% | 0,0% | 0,0% | 0,0% | 0,0% | 0,0% | 0,0% |  |  |  |  |
| Snijder_2021 | 0,0% | 0,0% | 0,0% | 0,0% | 0,0% | 0,0% | 0,0% | 0,0% | 0,0% | 0,0% | 0,0% | 0,0% | 0,0% | 0,0% | 0,0% | 0,0% |  |  |  |
| Vigni-Pau_2021 | 0,0% | 0,0% | 0,0% | 0,0% | 0,0% | 0,0% | 0,0% | 0,0% | 0,0% | 4,8% | 0,0% | 0,0% | 0,0% | 0,0% | 2,8% | 0,0% | 0,0% |  |  |
| Ward_2013 | 0,0% | 0,0% | 0,0% | 0,0% | 0,0% | 0,0% | 0,0% | 0,0% | 0,0% | 0,0% | 0,0% | 0,0% | 0,0% | 0,0% | 0,0% | 0,0% | 0,0% | 0,0% |  |
| Wesemann_2022 | 0,0% | 0,0% | 0,0% | 0,0% | 0,0% | 0,0% | 0,0% | 0,0% | 1,6% | 0,0% | 0,0% | 0,0% | 0,0% | 0,0% | 0,0% | 0,0% | 0,0% | 0,0% | 0,0% |

# **Supplementary Material 5 – Overlap of Included Primary Studies in the Systematic Reviews (GROOVE Heatmap; Pérez-Bracchiglion et al., 2022)**

1. We decided not to use the PRIOR checklist (Gates et al., 2022), as it is specifically designed for overviews of reviews of healthcare interventions, which is not the focus of our overview of reviews. Instead, we selected a checklist that is more appropriate for our research topic and that also incorporates the most relevant items from the PRIOR checklist**.**

   Reference: Gates M, Gates A, Pieper D, et al. (2022). Reporting guideline for overviews of reviews of healthcare interventions: development of the PRIOR statement. *BMJ*, 378:e070849. https://doi.org/10.1136/bmj-2022-070849 [↑](#footnote-ref-1)
